# Low-Dose Irradiation Differentially Impacts Macrophage Phenotype in Dependence of Fibroblast-Like Synoviocytes and Radiation Dose

**DOI:** 10.1155/2019/3161750

**Published:** 2019-08-14

**Authors:** Lisa Deloch, Jana Fuchs, Michael Rückert, Rainer Fietkau, Benjamin Frey, Udo S. Gaipl

**Affiliations:** Department of Radiation Oncology, Universitätsklinikum Erlangen, Friedrich-Alexander-Universität Erlangen-Nürnberg (FAU), Erlangen 91054, Germany

## Abstract

Rheumatoid arthritis (RA) is a multifactorial autoimmune disease whose main hallmark is inflammation and destruction of the joints. Two cell types within the synovium that play an important role in RA are fibroblast-like synoviocytes (FLS) and macrophages. The latter innate immune cells show a high plasticity in their phenotype and are central in inflammatory processes. Low-dose radiotherapy (LD-RT) with particularly a single dose of 0.5 Gy has been demonstrated to have a positive impact on pain, inflammation, and bone in inflamed joints. We now examined for the first time how LD-RT influences FLS and bone marrow-derived macrophages in co-culture systems of an experimental model of RA to reveal further mechanisms of immune modulatory effects of low and intermediate dose of ionizing radiation. For this, the bone marrow of h*TNF-α* tg mice was differentiated either with cytokines to obtain key macrophage phenotypes (M0, M1, and M2) or with supernatants (SN) of untreated or irradiated FLS. Flow cytometry analyses were used to analyse the impact of radiation (0.1, 0.5, 1.0, and 2.0 Gy) on the phenotype of macrophages in the presence or absence of SN of FLS. LD-RT had no impact on cytokine-mediated macrophage polarization in M0, M1, or M2 macrophages. However, SN of irradiated FLS particularly reduced CD206 expression on macrophages. Macrophage phenotype was stable when being in contact with SN of nonirradiated FLS, but significantly increased surface expression of CD206 and slightly decreased CD80 and CD86 expression were observed when macrophage themselves were irradiated with 0.5 Gy under these microenvironmental conditions, again highlighting discontinuous dose dependencies in the low and intermediate dose range. One can conclude that FLS-dependent microenvironmental conditions have a slight influence on the modulation of macrophage phenotype under radiation exposure conditions. Future studies are needed to reveal the impact of radiation exposure on the functions of treated macrophages under such microenvironmental conditions.

## 1. Introduction

Rheumatoid arthritis (RA) is a multifactorial autoimmune disease that is associated with inflammatory infiltration of the joints leading to an advancing destruction of the bone and cartilage accompanied by chronic inflammation [1, 2]. RA has a high prevalence among the world's population and thus is linked to high personal and socioeconomic costs [1, 3]. The exact cause of RA is still unknown, but the progression of the disease is in part mediated by infiltrating immune cells, fibroblast-like synoviocytes (FLS), and osteoclasts (OCs). While the latter are responsible for final bone destruction, FLS are considered to be the primary cell type involved in joint destruction as these cells contribute to disease initiation, progression, and maintenance of inflammation of the joints and secrete enzymes such as matrix metalloproteinases (MMPs) that digest the cartilage [4–8]. FLS further modulate inflammatory processes within the joints through the secretion of various cytokines and chemokines being responsible for the recruitment of lymphocytes and monocytes [9, 10]. Next to FLS, various effector cells such as macrophages are found in the synovial membrane, and cytokines, such as macrophage colony-stimulating factor (M-CSF) and granulocyte macrophage colony-stimulating factor (GM-CSF), are present. These factors further mediate the infiltration of the synovium and the maturation of immune cells, e.g., monocytes to macrophages.

Macrophages are central effectors of inflammation and tissue destruction in RA that mainly act through the release of soluble factors such as tumor necrosis factor-*α* (TNF-*α*) as well as interleukin- (IL-) 1 and IL-6, reactive oxygen species, and matrix degrading enzymes. Their numbers often correlate with disease activity in RA and successful anti-rheumatic treatment. Therefore, macrophages as well as their maturation factors, such as M-CSF and GM-CSF, are possible therapeutic targets [1, 11–14]. Inflammatory M1 macrophages are the predominant type within the synovium [1, 13, 15].

Macrophages can be divided into different subsets: the classical activated, proinflammatory M1 subset and the alternatively activated, anti-inflammatory M2 subset. However, several further subclasses can be defined. M1 activation is mediated by molecules that are associated with infectious microorganisms such as lipopolysaccharides (LPS) and inflammatory cytokines such as interferon- (IFN-) *γ*. M1 macrophages play an important role in the initiation and development of inflammatory events and produce a number of effector molecules such as reactive oxygen species (ROS), inducible nitric oxide synthase (iNOS), and inflammatory cytokines such as IL-1*β*, IL-6, and TNF-*α*. Additionally, M1 macrophages express activation markers such cluster of differentiation (CD)86 and CD80 as well as major histocompatibility complex (MHC)II. Due to their inflammatory properties, chronic activation of M1 macrophages can cause tissue damage [15–18]. M2 macrophages on the other hand are primed in response to Th2-related cytokines such as IL-4 and IL-10, and they express high levels of CD206, arginase 1, and anti-inflammatory cytokines. M2 macrophages can further be divided into various subtypes that all have their own set of markers to distinguish between them [15–18]. However, this M1/M2 classification is mainly feasible in cell culture experiments. *In vivo*, these main subtypes are not strictly formed, but are rather interchangeable. Growing evidence suggests that M1/M2 imbalances are connected to a number of diseases including RA [15, 17]. A positive correlation between the M1/M2 ratio and the number and bone resorbing activity of osteoclasts has been found [17], making macrophages a promising target for further treatment strategies of RA.

Even though the advanced understanding of the rheumatic diseases has led to better therapeutic options with improved outcome [1, 2], there are still patients that do not respond sufficiently [19]. This is mainly due to therapy resistance or serious side effects [13, 20], stressing the need for additional therapeutic options [2, 19, 20]. For these patients, a treatment with low/intermediate doses of X-rays (low-dose radiotherapy (LD-RT)) could be a beneficial alternative and/or supplement. It has been shown that LD-RT ameliorates existing inflammation and bone loss in degenerative musculoskeletal diseases [20–24]. Even though single fractions with a dose ranging from 0.5 to 1.0 gray (Gy) are applied, a single dose of 0.5 Gy/fraction with a total dose of 3 Gy per series has been shown to be as effective as 1.0 Gy in terms of pain reduction, but even better in ameliorating inflammation and reducing bone destruction [20, 21, 24–30]. Although a lot of the molecular mode of actions that lead to the anti-inflammatory effects of LD-RT are still unknown, it has become clear that ionizing radiation is able to reduce inflammation through various mechanisms, such as the induction of apoptosis in immune cells, reduced leukocyte adhesion, the secretion of anti-inflammatory soluble factors, and a reduced function of macrophages and positive effects on the bone metabolism [21, 31–35]. One has to stress that discontinuous dose response relationships are mostly seen in this dose range [30]. Nonetheless, most of these experiments on the impact of LD-RT on cells being present in the inflamed joints have been carried out with isolated cell types. However, it becomes more and more evident that heterotypic and homotypic cell-cell interactions are vital for their biological function [14, 36, 37]. Therefore, co-culture systems should be increasingly used to elucidate, e.g., the influence of secreted factors of one cell type on phenotypic changes of another cell type [36, 38].

Macrophages and FLS are both high in numbers in the synovial tissue, and their interaction is crucial for inflammation and tissue damage in RA. It has already been shown that contact between these cells is sufficient to initiate the production of a plethora of proinflammatory cytokines [14]. As we have already observed that macrophages are very radioresistant [31, 39] and LD-RT reduced the inflammatory phenotype of FLS in an experimental mouse model of RA (human *TNF-α* tg mice, strand tg197; h*TNF-α* tg) [28], we now aimed to investigate for the first time the effects of LD-RT in a conditioned medium system of FLS and bone marrow-derived macrophages in an experimental model of RA on macrophage phenotype. This should strengthen the knowledge about immune modulatory effects of low and intermediate doses of radiation and will have a potential impact on therapeutic applications of LD-RT for benign diseases in the future.

## 2. Materials and Methods

### 2.1. Mice

h*TNF-α* tg mice were kindly provided by Prof. George Kollias (Fleming Institute, Vari, Greece; MTA 842/18) and kept and maintained in a SPF facility under a sterile atmosphere at the animal facility of the Universitätsklinikum Erlangen, the Franz-Penzoldt-Centre (approval number 55.2-DMS-2532-2-114). All animal procedures have been approved by the *Regierung of Unterfranken* (approval number TS-3/14) and were conducted in accordance within the guidelines of the Federation of European Laboratory Animal Science Associations (FELASA).

### 2.2. Fibroblast-Like Synoviocyte Cultures

Fibroblast-like synoviocytes (FLS) were prepared in accordance with the protocol of Armaka et al. [40] and as previously described in our work on how LD-RT ameliorates advanced arthritis in h*TNF*-*α* tg mice [28]: hind paws of h*TNF*-*α* tg mice were prepped and digested in a 1% collagenase type IV solution (Gibco, Carlsbad, CA, USA) on a shaker (37°C, 1400 rpm). Isolated cells were kept at standard culture conditions (37°C, 5% CO_2_; 95% humidity) in medium containing 50% Dulbecco's modified Eagle medium (DMEM; Pan Biotech, Aidenbach, Germany) and 50% F-12 medium (Gibco) supplemented with 10% fetal bovine serum (FBS; Biochrom, Berlin, Germany), 1% penicillin/streptomycin (PS; Gibco), and 1% low serum growth supplement (LSGS; Gibco). The purity of the cells was determined at passage 5 using flow cytometry. FLS were considered to be CD11b^−^, CD54^+^, and CD106^+^ (see Table 1 for antibodies and Supplementary Figure 1 for exemplary phenotyping). In total, three independent FLS pools were used. Supernatants (SN) of irradiated and untreated FLS cultures were collected and stored at -80°C until they were added to macrophage cultures.

### 2.3. Macrophage Differentiation

**Figure 1:**
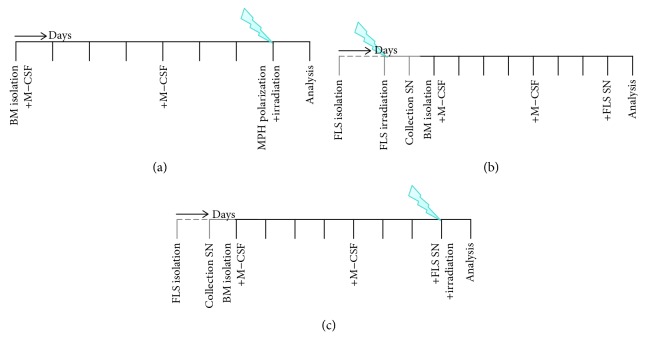
Visualization of experimental *in vitro* co-culture and radiation set-up. Experimental set-up for the assessment of cytokine-mediated (a) or supernatant- (SN-) mediated (b, c) macrophage polarization and the impact of LD-RT on it are displayed. In brief, first, bone marrow-derived macrophages were generated and polarized using cytokines only (a). For the following experiments (b, c), prior to macrophage generation, fibroblast-like synoviocyte (FLS) cultures were generated and SN were collected from either irradiated (B) or untreated (C) FLS cultures and stored at -80°C. For experimental set-ups (a) and (c), macrophages were irradiated 2 h after the addition of polarization medium, and for experimental set-up (b), macrophages were treated with SN of irradiated FLS only. 24 h after the addition of polarization media ± irradiation, macrophage phenotypes were analysed.

Bone marrow-derived macrophages were isolated from the long bones of h*TNF-α* tg mice and stimulated with 5 ng/ml M-CSF (Miltenyi Biotec, Bergisch Gladbach, Germany) (d0) for 6 days with regular medium changes (Figure 1). On day 7, wells were subdivided into three groups and either stimulated with 5 ng/ml M-CSF for M0; 4 ng/ml GM-CSF (Miltenyi Biotec), 20 ng/ml interferon-*γ* (IFN-*γ*, ImmunoTools, Friesoythe, Germany), and 20 ng/ml lipopolysaccharide (LPS) for M1; and 5 ng/ml M-CSF and 20 ng/ml interleukin- (IL-) 4 (ImmunoTools) for M2 macrophages (Figure 1(a)) or stimulated with SN of FLS for 24 h, respectively (Figures 1(b) and 1(c)). Two hours after the addition of cytokines or SN, cells were irradiated (Figures 1(a) and 1(c)). Figure 1 summarizes these experimental set-ups. Cells were kept under standard conditions (37°C, 5% CO2; 95% humidity) in a medium containing Dulbecco's modified Eagle medium (DMEM, PAN Biotech, Aidenbach, Germany) supplemented with 10% fetal bovine serum (FBS, Biochrom AG, Berlin, Germany), 5% horse serum (Sigma-Aldrich, Darmstadt, Germany), 1% penicillin/streptomycin (Gibco), and 50 *μ*M 2-mercaptoethanol (Gibco).

### 2.4. *Ex Vivo* Irradiation of Cell Cultures

*Ex vivo* irradiation of macrophage and FLS cell cultures was carried out using a 120 keV Isolvolt Titan X-ray tube equipped with a 0.5 mm copper filter. Amperage (mA) and time were set according to the applied doses (0.1–2 Gy). The cell culture plates were placed on top of a Plexiglas® plate for better distribution of X-rays.

### 2.5. Flow Cytometry Analyses

For flow cytometry analyses, FLS were dissociated using *trypsin*, whereas macrophages were detached using cold PBS (Gibco, Gaithersburg, USA). Afterwards, the cells were resuspended in 2% FBS/PBS. Following a blocking step at room temperature, saturated antibodies in the indicated concentrations (Table 1) were added to the cell suspension and incubated at 4°C for 30 min. A Gallios flow cytometer (Beckman Coulter, Brea, CA, USA) was used for FLS phenotyping and a CytoFlex S (Beckman Coulter) for macrophage surface marker expression analyses. Used antibodies and their respective dilutions and application are summarized in Table 1. Gating strategy for FLS phenotyping is displayed in Supplementary Figure 1, and the one for macrophage identification is shown in Supplementary Figure 2.

### 2.6. Quantitative PCR Analyses

For further macrophage phenotyping, qPCR analyses were carried out. Total RNA from macrophage cultures was isolated using TriFast (peqlab, Darmstadt, Germany) and phenol-chloroform extraction, and 0.5 *μ*g RNA was transcribed into cDNA using a reverse transcription kit by Qiagen (QuantiTect®, Hilden, Germany) by following the manufacturer's recommendations. qPCR then was conducted with a Thermo Scientific (Waltham, MA, USA) SYBR Green kit. Normalization of qPCR measurements was performed using two housekeepers (Table 2). Bio-Rad primers (Table 2, Bio-Rad Laboratories, Inc., Hercules, CA, USA) were used according to the manufacturer's instructions.

### 2.7. Statistical Analyses

Graph generation and statistical analysis were performed using the GraphPad Prism software (GraphPad Software Inc.). All data are presented as the mean ± SEM. A nonparametric two-tailed Mann-Whitney *U* test was used in comparison to respective untreated controls unless specified otherwise. Significances were indicated as follows: ^∗^ *p* < 0.05, ^∗∗^ *p* < 0.01, and ^∗∗∗^ *p* < 0.001.

## 3. Results

### 3.1. LD-RT Does Not Alter Cytokine-Mediated Macrophage Polarization

**Figure 2:**
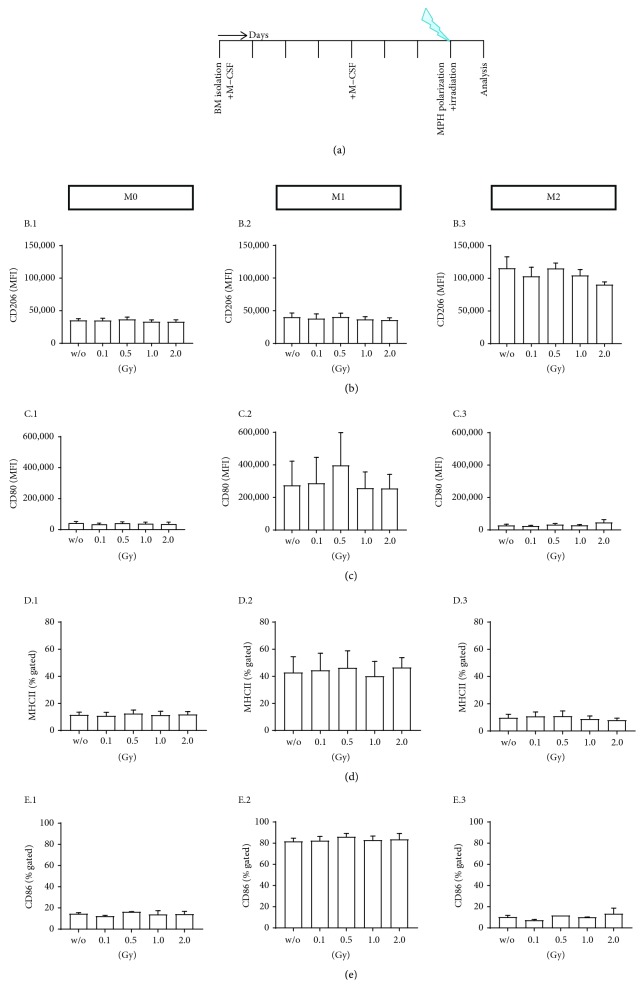
Low-dose radiotherapy has no influence on macrophage polarization. h*TNF-α* tg bone marrow was isolated and differentiated into M0 macrophages according to the experimental set-up shown in (a). 24 h prior to the characterization of the macrophage phenotypes, cells were treated with three different polarization cocktails (M0: 5 ng/ml M-CSF; M1: 4 ng/ml M-CSF, 20 ng/ml IFN-*γ*, and 20 ng/ml LPS; M2: 5 ng/ml M-CSF and 20 ng/ml IL-4). 2 h after this stimulation, cells were irradiated with the indicated dose. Macrophages showed characteristic surface marker expression after polarization such as CD206 ((b) B.1–B.3) for M2 and CD80 ((c) C.1–C.3), MHCII ((d) D.1–D.3), and CD86 ((e) E.1–E.3) for M1. No significant differences of the surface marker expression were found in dependence of irradiation and irradiation doses. Depicted is data from three independent experiments. Data is presented as mean mean ± SEM.

First, we tested whether LD-RT impacts cytokine-mediated macrophage polarization into M0, M1, and M2 macrophages, respectively. For this, h*TNF-α* tg bone marrow cells were stimulated with M-CSF for 7 days and 24 h before analysing the macrophage phenotype; the cells were treated with three different cytokine cocktails: M-CSF only was continuatively added to the culture (M0 macrophages); a cocktail that consisted of IFN-*γ*, GM-CSF, and LPS was used for generation of M1 macrophages; M-CSF and IL-4 were used for generation of M2 macrophages. Two hours after the addition of cytokines/LPS, cells were irradiated with various doses of X-rays (0, 0.1, 0.5, 1.0, and 2.0 Gy). The experimental set-up is displayed in Figure 2(a).

Macrophage subtypes could be identified according to their expression of characteristic surface markers (Figure 2; for gating strategy, see Supplementary Figure 2): M0 macrophages were specific neither for M1 nor for M2 markers (MFI of CD206 expression: 35133.49 ± 4642.90, Figure 2(b) B.1; CD80: 42466.78 ± 16937.31, Figure 2(c) C.1; MHCII: 11.54 ± 3.54%, Figure 2(d) D.1; CD86: 14.66 ± 1.28%, Figure 2(e) E.1). M1 macrophages showed particularly high expression levels of CD80 (274736.4 ± 257658.05, Figure 2(c) C.2), MHCII (42.83 ± 20.6%, Figure 2(d) D.2), and CD86 (81.8 ± 5.11%, Figure 2(e) E.2), while CD206 expression levels were similar to those of M0 macrophages (40230.28 ± 10939.32, Figure 2(b) B.2). M2 macrophages showed high expression levels of CD206 (115424.38 ± 30399.04, Figure 2(b) B.3), while typical M1 markers such as CD80 (27763.78 ± 13740.34, Figure 2(c) C.3), MHCII (9.76 ± 4.34%, Figure 2(d) D.3), and CD86 (10.19 ± 2.44%, Figure 2(e) E.3) were expressed at low levels. In addition to these surface markers, macrophage subpopulations were also characterized by qPCR analyses: M2-stimulated macrophages had higher Arg1 expression, whereas M1-polarized macrophages had higher Nos2 and TNF-*α* expression (Supplementary Figure 3). No significant influence of LD-RT on macrophage polarization was found, as characteristic surface molecule expression of M0, M1, and M2 macrophages remained stable following irradiation (Figures 2(b)–2(e)).

### 3.2. SN of Irradiated FLS Alter Macrophage Phenotype

**Figure 3:**
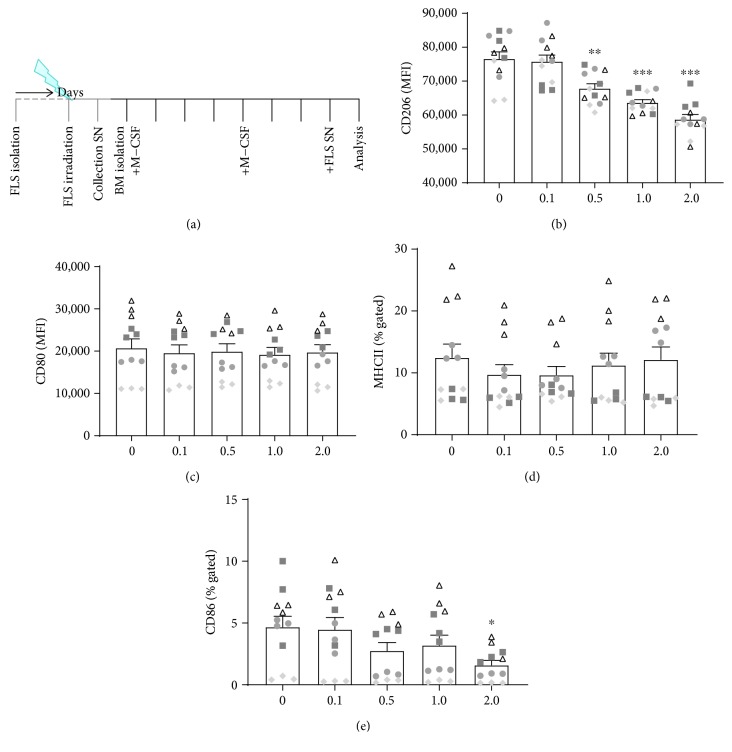
Conditioned supernatants of irradiated fibroblast-like synoviocytes have an impact on the expression of phenotype characteristic surface markers of macrophages in dependence of the radiation dose. h*TNF-α* tg bone marrow cells were isolated and differentiated into M0 macrophages according to the experimental set-up shown in (a). Prior to macrophage generation, fibroblast-like synoviocyte cultures (FLS) were generated, phenotyped, and irradiated. Conditioned SN were collected and stored at -80°C until they were added to macrophage cultures 24 h prior to characterization of the macrophage phenotype. The M2 marker CD206 (b) was significantly reduced at doses starting from 0.5 Gy while M1 markers CD80 (c), MHCII (d), and CD86 (e) showed fewer alterations. While CD80 expression was not influenced by LD-RT, MHCII showed a slight reduction at 0.1 and 0.5 Gy, respectively. CD86 expression was significantly reduced at 2.0 Gy and already slightly downregulated at 0.5 and 1.0 Gy. Depicted is data from four independent experiments, each performed in triplicate (data points from one experiment are marked with equal symbols). Data is presented as the mean ± SEM. ^∗^ *p* < 0.05, ^∗∗^ *p* < 0.01, and ^∗∗∗^ *p* < 0.001. Representative histograms of the analyses are displayed in Supplementary Figure 4A.

In a previous work, we already revealed that low doses of X-rays directly impact inflammatory FLS phenotype in a rather anti-inflammatory manner [28]. We were next interested whether the macrophage phenotype can be influenced by radiation during their polarization, determined by environmental condition such FLS. We therefore tested whether conditioned SN of nonirradiated or irradiated FLS also impact on the macrophage phenotype. Therefore, SN of FLS cultures that were either non-irradiated (0) or irradiated with low and intermediate doses of X-ray (0.1, 0.5, 1.0, and 2.0 Gy) were collected and added to M-CSF-differentiated M0 macrophages for 24 h prior to characterization of the macrophage phenotype (Figure 3(a)).

Conditioned SN of FLS induced a polarization of macrophages in a mixed phenotype, compared to cytokine cocktail-induced key M1/M2 macrophage subtypes (Figures 3(b)–3(e)). Expression of CD206 was approximately two-fold higher compared to M0 (2.18-fold) and M1 (1.9-fold) macrophages, but only 66% of that of the pure M2 macrophage population after stimulation with SN of nonirradiated FLS. Further, SN of irradiated FLS induced a significant, dose-dependent decrease in CD206 surface expression starting at a dose of 0.5 Gy (Figure 3(b)). CD80 surface expression was generally found to be lower than that in pure macrophage populations (only 49% of the expression level in M0, 8% of that in M1, and 75% of that in M2 macrophages; Figure 3(c)). However, SN of irradiated FLS did, in contrast to CD206, not significantly alter the surface expression of CD80 on macrophages. MHCII expression of FLS SN-stimulated macrophages was similar to that observed in M0 (1.08-fold), but lower compared to pure M1 macrophages (29%) and slightly increased compared to that of M2 macrophages (1.28-fold) (Figure 3(d)). SN of irradiated FLS had a slight impact on MHCII surface expression when being collected from FLS that had been irradiated with 0.1 and 0.5 Gy, respectively. CD86 surface expression was reduced compared to cytokine-stimulated macrophage subcultures (32% of that of M0, 6% of that of M1, and 46% of that of M2 macrophages; Figure 3(e)). SN of irradiated FLS were added to that effect by slightly reducing CD86 expression on a small subpopulation of macrophages (around 4%) following incubation of macrophages with SN of FLS that had been irradiated with 0.5 or 1.0 Gy and significantly after 2.0 Gy (to around 2%).

### 3.3. Irradiation with 0.5 Gy of FLS SN-Stimulated Macrophages Induces a More M2-Like Macrophage Phenotype

**Figure 4:**
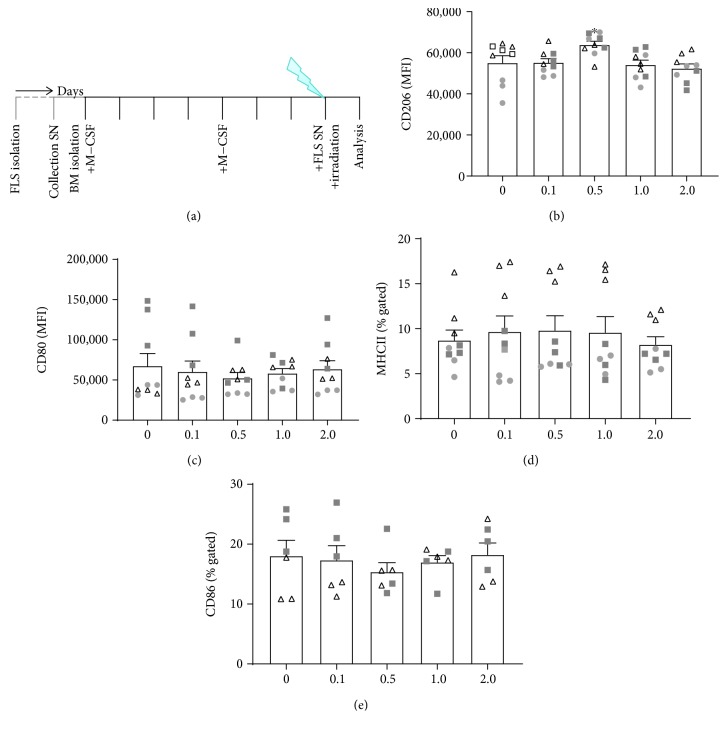
Irradiation with 0.5 Gy alters fibroblast-like synoviocyte supernatant-stimulated macrophage phenotype. h*TNF*-*α* tg bone marrow cells were isolated and differentiated with 5 ng/ml M-CSF into M0 macrophages according to the experimental set-up shown in (a). Prior to macrophage generation, fibroblast-like synoviocyte (FLS) cultures were generated and phenotyped. SN of FLS were collected and stored at -80°C until they were added to macrophage cultures 24 h prior to characterization of the macrophage phenotype and 2 h prior to irradiation with the displayed doses of X-rays. The M2 macrophage marker CD206 (b) was significantly increased after irradiation with 0.5 Gy. M1 macrophage markers CD80 (c) and CD86 (e) showed a slightly reduced surface expression again after irradiation with 0.5 Gy, while the expression of MHCII (D) was not altered. Depicted is data from three independent experiments, each performed in triplicate (data points from one experiment are marked with equal symbols). Data is presented as the mean ± SEM. ^∗^ *p* < 0.05. Representative histograms of the analyses are displayed in Supplementary Figure 4B.

We lastly focussed on whether irradiation itself has an impact on polarization of M0 macrophages that are in contact with SN of FLS (Figure 4(a)). As already mentioned above, conditioned SN of FLS induced a polarization of macrophages in a mixed phenotype, compared to cytokine cocktail-induced key M1/M2 macrophage subtypes. Irradiation of FLS SN-stimulated macrophages induced a significantly increased expression of CD206, but only at 0.5 Gy (Figure 4(b)). Regarding the expression of CD80, the most prominent, but not significant, reduced expression was observed after irradiation with 0.5 Gy (Figure 4(c)). Similar effects were observed for the expression of CD86 (Figure 4(e)). The expression of MHCII was not altered by irradiation (Figure 4(d)).

## 4. Discussion

FLS and macrophages are both abundant within the synovial tissue. The interplay of these cell types results in microenvironmental conditions that substantially contribute to tissue damage and inflammation in RA [14]. Modulation of these cell types might therefore be promising for the treatment of RA, such as a reduction of proinflammatory M1 macrophages that are the predominant macrophage phenotype in the synovium [1, 13, 15]. As LD-RT has already been demonstrated to ameliorate inflammation and to reduce bone destruction, we here analysed for the first time its impact on both macrophages and FLS.

Since we observed that already polarized macrophages are stable in their phenotype one day after LD-RT (Figure 2), we were next interested whether the macrophage phenotype can be influenced by radiation during their polarization that is determined by environmental condition such as SN of FLS (Figure 3). This would confirm that interactions between ionizing radiation and the immune system are very complex and multifaceted and depend on both, the irradiated cells and the microenvironment [41–43]. The nature of the irradiated tissues (cell type, microenvironment, and inflammation status) might strongly pretend the radiation response [44]. Our experiments revealed that irradiation with a single dose of 0.5 Gy of macrophages that had been in contact with SN of inflammatory FLS changes the phenotype of the macrophages to a more anti-inflammatory manner. Significantly increased expression of CD206 and slightly reduced expression of CD80 and CD86 were only seen after exposure to 0.5 Gy (Figure 4(b)). This strengthens the fact that within the low and intermediate dose range, a biphasic dose response is often observed. Such discontinuous dose-response relationships have been demonstrated in various settings and cell types [26, 28, 29, 31, 32, 45, 46].

Since CD206 expression was reduced in non-irradiated macrophages (Figure 3(b)), but enhanced on the ones that were exposed to 0.5 Gy (Figure 4(b)), first hints exist that macrophages that do infiltrate after the tissue was irradiated may polarize differently than those macrophages that were present at the time of irradiation. One can therefore hypothesise that infiltrating macrophages that enter the synovium after the irradiation process have a less anti-inflammatory phenotype. Future experiments should additionally elucidate whether functional differences do exist between resident and recruited macrophages.

A LD-RT-induced shift towards a more M2-like macrophage phenotype might contribute to the reduction of RA symptoms [15]. Even though SN of irradiated FLS resulted in a slight decreased subpopulation of macrophages expressing CD86 (from around 4 to around 2%), it also decreased the expression of CD206 (Figure 3). Since irradiation also impacts on many other factors, this small downregulation of CD86 is expected to have physiological relevance only in combination with other radiation-induced alterations of the macrophage phenotype.

This highlights several facts:

- Macrophages do have a high plasticity also under irradiation conditions, meaning that similar as in non-irradiation exposure situations, the phenotype of these immune cells is very flexible. This confirms that, under inflammatory conditions and when being exposed to radiation, functional polarization of macrophages into only two groups is an oversimplified description and it is necessary to consider a continuum of functional states [47]
- It is of great importance for the final immunological outcome which cell type is predominantly irradiated (targeted by irradiation) and in which microenvironmental conditions the irradiation takes place
- Resident and infiltrating macrophages might react differently to a local irradiation. Infiltrating macrophages that enter the synovium after the irradiation process should have a less anti-inflammatory phenotype
- By exploiting the beneficial therapeutic effects of ionizing radiation on the immune response, the model system and interactions of different cell types have to be strongly considered
- Discontinuous dose-effect relationships are frequently in immunological events, as it has already been demonstrated for several scenarios before [46, 48]

We found no significant influences of LD-RT on cytokine-stimulated macrophage subcultures, suggesting a strong phenotypic stability of pre-differentiated macrophages and a high radioresistance of these innate immune cells. This is in accordance with previous findings that viability of macrophages is not influenced by LD-RT [31, 33, 39, 49]. However, when conditioned media of FLS cultures were present, alternating effects of LD-RT on macrophage polarity in dependence of the experimental set-up were found (Figures 3 and 4). This is in accordance with a co-culture experiment of mouse fibroblasts and macrophages that revealed that even though a combination of the two cell types resulted in significant cartilage degradation, the effects of separated cell cultures were much less pronounced [50].

In this first study, on how radiation impacts on macrophage polarization, we focussed on the macrophage phenotype rather than functionality. We therefore proceeded accordingly to the already established protocols where macrophages were exposed to cytokines for 24 h in order to generate M1/M2 subtypes [51, 52]. We aimed to provide a direct comparison between cytokine- and supernatant-stimulated macrophages. Nevertheless, a future work should additionally focus on delayed functional effects of low dose exposure as already observed for other immune biological settings [53].

## 5. Conclusion

Our work indicates that, under inflammatory conditions, macrophages can be altered in their phenotype by radiation; however, this is strongly dependent on the microenvironment. This result stresses the fact that not only a particular radiation dose but also certain environmental conditions have an impact on immune alterations. Immune cells do rarely act on their own *in vivo* but mainly depend on interactions with other cell types and secreted factors; this should always be kept in mind when designing *in vitro* experiments for following up the influence of ionizing radiation on the immune system and parts of it.

We revealed that macrophages do have high plasticity also under irradiation conditions, meaning that similar as in non-radiation exposure situations, the phenotype of these immune cells is very flexible. This confirms that, under inflammatory conditions and when being exposed to radiation, functional polarization of macrophages into only two groups is an oversimplified description and it is necessary to consider a continuum of states. We want to stress that we here present the first data that give hints that radiation in the low and intermediated dose range impacts the macrophage phenotype in consecutive action with the microenvironment. The observed effects, based on classical activation and differentiation markers of macrophages, however do only provide the first hints that radiation is involved in macrophage plasticity. We further confirmed the macrophage phenotypes by analysing the expression Arg1, Nos2, and TNF-*α* by qPCR (Supplementary Figure 3). Nevertheless, further studies are needed to reveal the impact of radiation exposure on the functions of these treated macrophages. It has been shown before, even though irradiation of macrophages results in DNA damage [54], macrophages remain viable and metabolically active [31, 54]. Nevertheless, they do show alterations regarding their cytokine profile and in chemotaxis [31]. While our model systems used in this work suggest a positive influence of radiation exposure on macrophage polarization in the inflamed synovial tissue, when FLS and macrophages are both exposed to irradiation, which is also the case in patients whose joints are treated with LD-RT, we are still aiming for a better understanding of their functional properties in future experiments. Combined with data of previously published works [28, 29], alongside with the known beneficial effects of LD-RT on degenerative, inflammatory joint diseases [24, 25], we postulate an overall beneficial effect of LD-RT for such patients by radiation-induced alterations of the immune microenvironment.

## Figures and Tables

**Table 1:** Antibodies used for flow cytometry-based FLS and macrophage phenotyping.

Antibody	Dilution	Manufacturer	Application
CD11b-FITC	1 : 100	BD Bioscience, Franklin Lakes, NJ, USA	FLS phenotyping
CD106/VCAM-1-FITC	1 : 100	eBioscience, Frankfurt, Germany	FLS phenotyping
CD54/ICAM-1-PE	1 : 400	eBioscience	FLS phenotyping
IgG2b*κ*-FITC	1 : 100	BD Bioscience	FLS phenotyping
IgG2a*κ*-FITC	1 : 100	BD Bioscience	FLS phenotyping
IgG2a*κ*-PE	1 : 400	BD Bioscience	FLS phenotyping
CD11b-FITC	1 : 500	BD Bioscience	Macrophage characterization
F4/80-eFluor660	1 : 750	eBioscience	Macrophage characterization
MHCII-eFluor450	1 : 1000	eBioscience	Macrophage characterization
CD80-PE	1 : 500	BD Bioscience	Macrophage characterization
CD86-perCP-Vio700	1 : 50	Miltenyi Biotec, Bergisch Gladbach, Germany	Macrophage characterization
CD206-BV605	1 : 20	BioLegend, San Diego, CA, USA	Macrophage characterization

**Table 2:** PCR primers used for characterization of macrophage polarization.

Gene	Primer	Unique assay ID	Application
Actin, beta	Actb	qMmuCED0027505	Housekeeper
Arginase	Arg1	qMmuCID0022400	M2
Tumor necrosis factor-*α*	TNF-*α*	qMmuCED0004141	M1
Nitric oxide synthase 2, inducible	Nos2	qMmuCID0023087	M1
Ribosomal protein S18	Rps18	qMmuCED0045430	Housekeeper

## Data Availability

The data used to support the findings of this study are available from the corresponding author upon request.

## Supplementary Materials

Supplementary Materials Supplementary Figure 1: flow cytometry-based gating strategy for FLS phenotyping. FLS isolated from the hind paws of h*TNF*-*α* tg mice was cultivated until passage 5 before phenotyping. The latter was carried out using flow cytometry analysis, whereas cells such as CD11b^−^ (A), CD54^+^ (B), and CD106^+^ (C) were considered to be FLS. Unstained and isotype-stained samples served as controls. Depicted is exemplary data (histogram overlay) from one h*TNF*-*α* tg-derived FLS cell pool. Supplementary Figure 2: flow cytometry-based gating strategy for the analysis of macrophage polarization. Bone marrow cells of the long bones of h*TNF*-*α* tg mice were isolated and differentiated with 5 ng/ml M-CSF with frequent medium changes. 24 h before harvest, cells were treated with cytokine cocktails for differentiation into polarized macrophages. Afterwards, the phenotype of the macrophages was determined by multicolour flow cytometry. After the exclusion of duplets (A), viable cells were gated according to their FSC/SSC properties (B) and macrophages were consecutively identified as being positive for F4/80 and CD11b(C). (D)–(G) show fluorescent minus one (FMO) stainings for gate placement of macrophage phenotype analyses. Here, pregated macrophages (C) were further examined with regard to the respective pro- and anti-inflammatory surface markers MHCII (D), CD86 (E), CD80 (F), and CD206 (G), respectively. Supplementary Figure 3: flow cytometry- and quantitative PCR- (qPCR-) based characterization of macrophage cultures. Bone marrow cells from the long bones of h*TNF*-*α* tg mice were isolated and differentiated with 5 ng/ml M-CSF with frequent medium changes. 24 h before harvest, cells were treated with cytokine cocktails for differentiation into M0- (5ng/ml M-CSF), M1- (4 ng/ml GM-CSF; 20 ng/ml IFN-*γ*; and 20 ng/ml LPS), or M2- (5ng/ml M-CSF; 20 ng/ml IL4) polarized macrophages. (A)–(D) show flow cytometry-based and (E)–(G) qPCR-based identification of macrophage phenotypes, respectively. M2-stimulated macrophages were considered to be CD206^+^ (A) as well as Arg1^+^ (E) whereas M1-polarized ones where considered to be CD80^+^ (B), MHCII^+^ (C), CD86^+^ (D), Nos2^+^ (F), and *TNF*-*α* ^+^ (G). Supplementary Figure 4: exemplary histograms of the flow cytometry-based analyses of expression of CD206 macrophage surface marker. Bone marrow cells from the long bones of h*TNF*-*α* tg mice were isolated and differentiated into M0 macrophages. Prior to macrophage generation, fibroblast-like synoviocyte cultures (FLS) were generated, phenotyped, and partly irradiated. Conditioned SN of irradiated and nonirradiated FLS cultures were collected and stored at -80°C until they were added to macrophage cultures 24 h prior to characterization of the macrophage phenotype. Shown is an exemplary experiment that visualizes the decreased surface expression of CD206 starting from 0.5 Gy when macrophages are in contact with SN of irradiated FLS (A) and the slightly increased surface expression of CD206 when macrophages that had been in contact with SN of FLS are irradiated with 0.5 Gy (B).
