# Supplementary material for: Low-Dose Irradiation Differentially Impacts Macrophage Phenotype in Dependence of Fibroblast-Like Synoviocytes and Radiation Dose
Source: J Immunol Res. 2019 Aug 14;2019:3161750. doi: 10.1155/2019/3161750 (PMC6710796; doi:10.1155/2019/3161750)
Supplement: Supplementary Materials — Supplementary Figure 1: flow cytometry-based gating strategy for FLS phenotyping. FLS isolated from the hind paws of hTNF-α tg mice was cultivated until passage 5 before phenotyping. The latter was carried out using flow cytometry analysis, whereas cells such as CD11b− (A), CD54+ (B), and CD106+ (C) were considered to be FLS. Unstained and isotype-stained samples served as controls. Depicted is exemplary data (histogram overlay) from one hTNF-α tg-derived FLS cell pool. Supplementary Figure 2: flow cytometry-based gating strategy for the analysis of macrophage polarization. Bone marrow cells of the long bones of hTNF-α tg mice were isolated and differentiated with 5 ng/ml M-CSF with frequent medium changes. 24 h before harvest, cells were treated with cytokine cocktails for differentiation into polarized macrophages. Afterwards, the phenotype of the macrophages was determined by multicolour flow cytometry. After the exclusion of duplets (A), viable cells were gated according to their FSC/SSC properties (B) and macrophages were consecutively identified as being positive for F4/80 and CD11b(C). (D)–(G) show fluorescent minus one (FMO) stainings for gate placement of macrophage phenotype analyses. Here, pregated macrophages (C) were further examined with regard to the respective pro- and anti-inflammatory surface markers MHCII (D), CD86 (E), CD80 (F), and CD206 (G), respectively. Supplementary Figure 3: flow cytometry- and quantitative PCR- (qPCR-) based characterization of macrophage cultures. Bone marrow cells from the long bones of hTNF-α tg mice were isolated and differentiated with 5 ng/ml M-CSF with frequent medium changes. 24 h before harvest, cells were treated with cytokine cocktails for differentiation into M0- (5ng/ml M-CSF), M1- (4 ng/ml GM-CSF; 20 ng/ml IFN-γ; and 20 ng/ml LPS), or M2- (5ng/ml M-CSF; 20 ng/ml IL4) polarized macrophages. (A)–(D) show flow cytometry-based and (E)–(G) qPCR-based identification of macrophage phenotypes, respectively. M2-stimul [file 3161750.f1.pdf]

Supplementary Materials

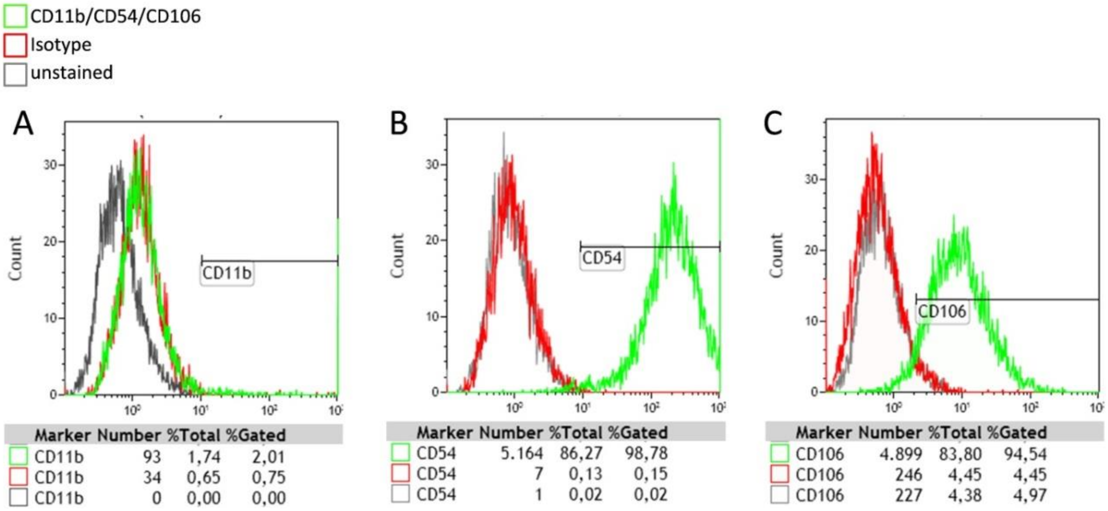

Supplementary Figure 1: Flow cytometry-based gating strategy for FLS phenotyping

FLS isolated from the hind paws of h*TNF-α* tg mice were cultivated until passage 5 before phenotyping. The latter was carried out using flow cytometry analysis, whereas cells that were $CD11b^{-}$ (A), $CD54^{+}$ (B), and $CD106^{+}$ (C) were considered to be FLS. Unstained and isotype-stained samples served as controls. Depicted is exemplary data (histogram overlay) for one h*TNF-α* tg-derived FLS cell pool.

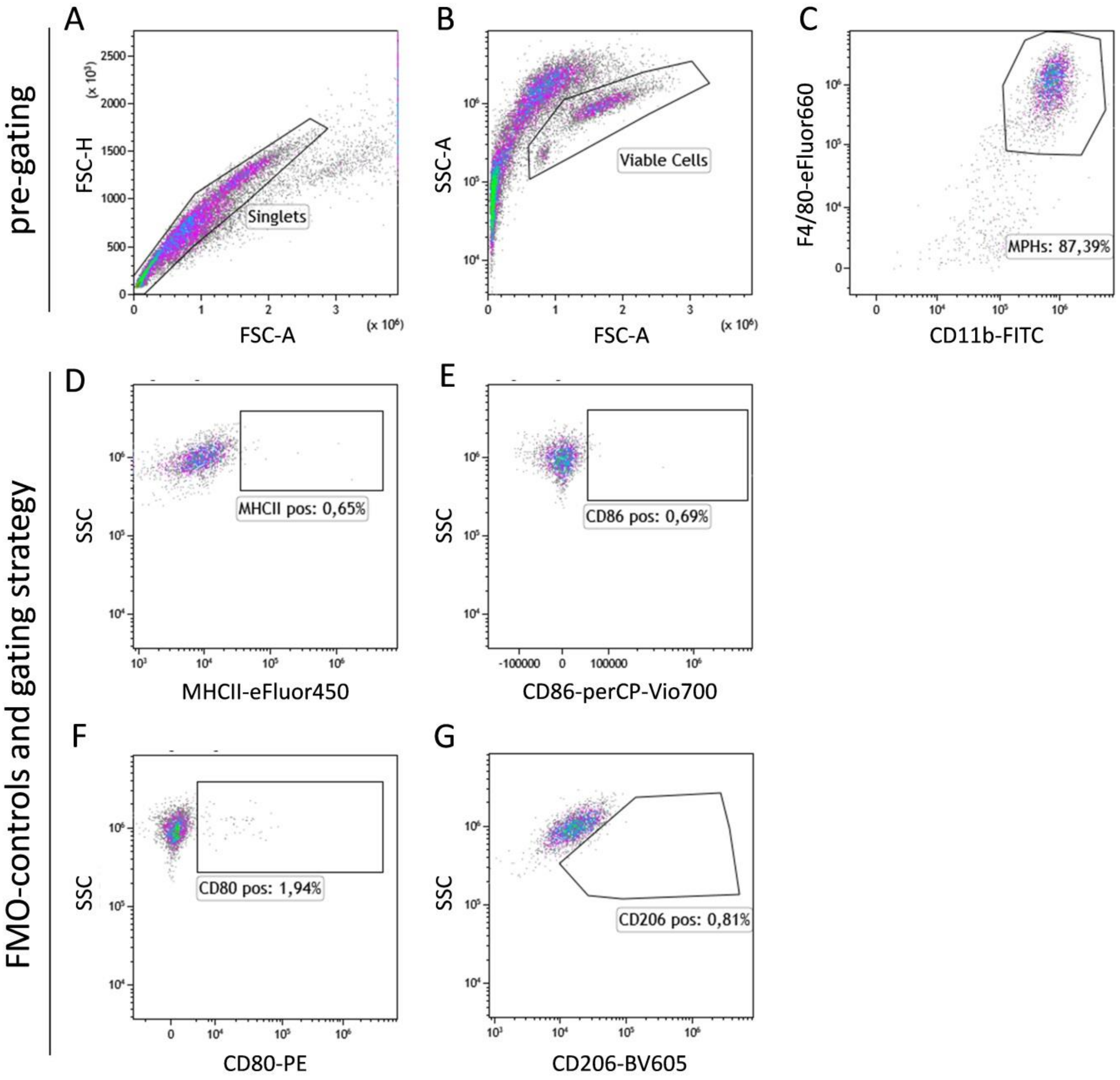

Supplementary Figure 2: Flow cytometry-based gating strategy for analysis of macrophage polarization
Bone marrow cells of the long bones of h*TNFα* tg mice were isolated and differentiated with 5ng/ml M-CSF with frequent media changes. 24h before harvest, cells were treated with cytokine cocktails for differentiation into polarized macrophages. Afterwards, the phenotype of the macrophages was determined by multicolour flow cytometry. After exclusion of duplets (A), viable cells were gated according to their FSC/SSC properties (B) and macrophages were consecutively identified as being positive for F4/80 and CD11b(C). D-G show *fluorescent minus one* (FMO) stainings for gate placement of macrophage phenotype analyses. Here, pre-gated macrophages (C) were further examined with regards to the respective pro- and anti-inflammatory surface markers MHCII (D), CD86 (E), CD80 (F), and CD206 (G), respectively.

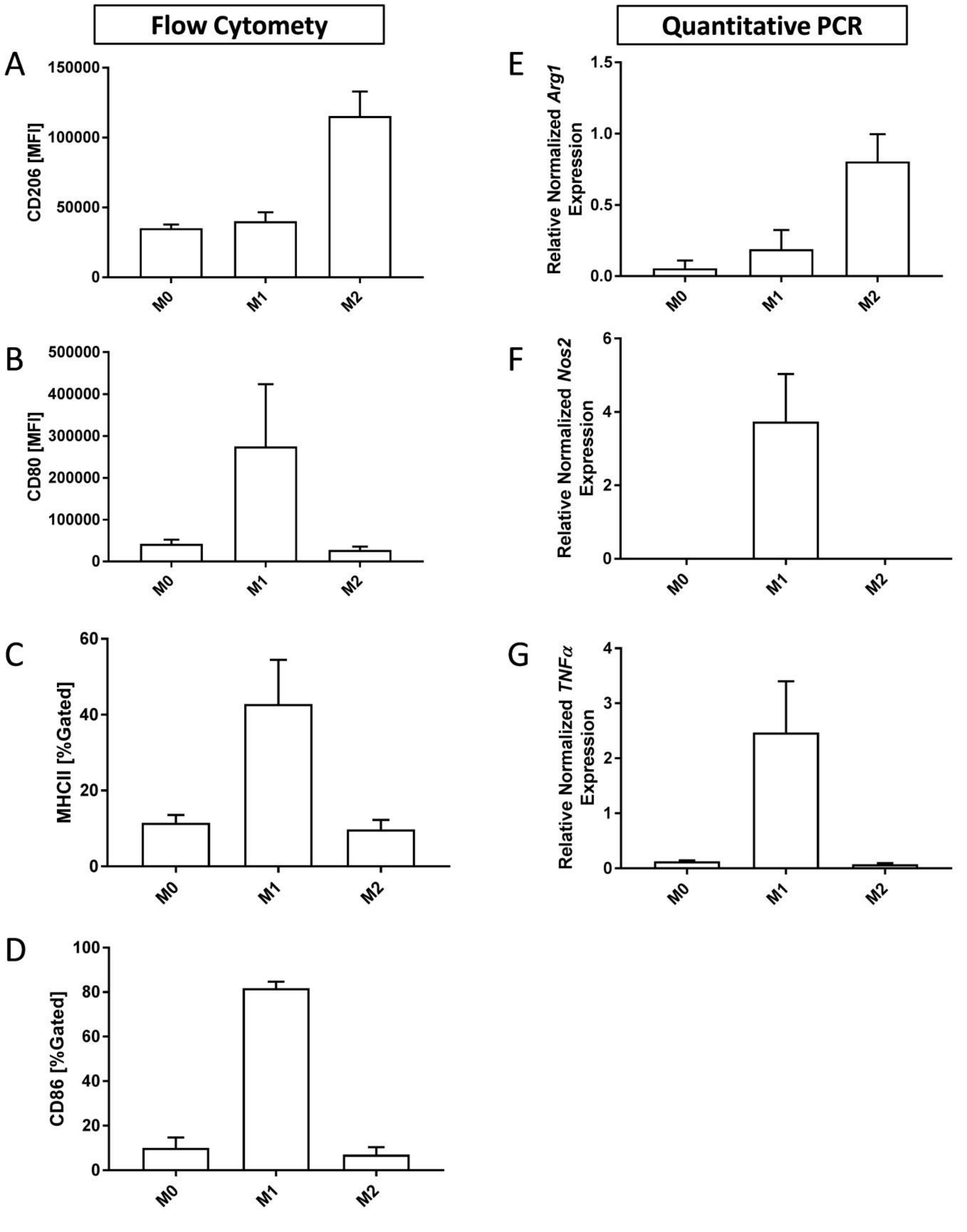

Supplementary Figure 3: Flow cytometry- and quantitative PCR (qPCR)-based characterization of macrophage cultures

Bone marrow cells from the long bones of h*TNFα* tg mice were isolated and differentiated with 5ng/ml M-CSF with frequent media changes. 24h before harvest, cells were treated with cytokine cocktails for differentiation into M0 (5ng/ml M-CSF), M1 (4ng/ml GM-CSF; 20ng/ml IFNγ; 20ng/ml LPS), or M2 (5ng/ml M-CSF; 20ng/ml IL4) polarized macrophages. A-D show flow cytometry-based and E-G qPCR-based identification of macrophage phenotypes, respectively. M2 stimulated macrophages were considered to be $CD206^{+}$ (A) as well as *Arg1*+ (E) whereas, M1 polarized ones where considered to be $CD80^{+}$ (B), $MHCII^{+}$ (C), $CD86^{+}$ (D), *Nos2*$^{+}$ (F), and *TNFα*$^{+}$ (G).

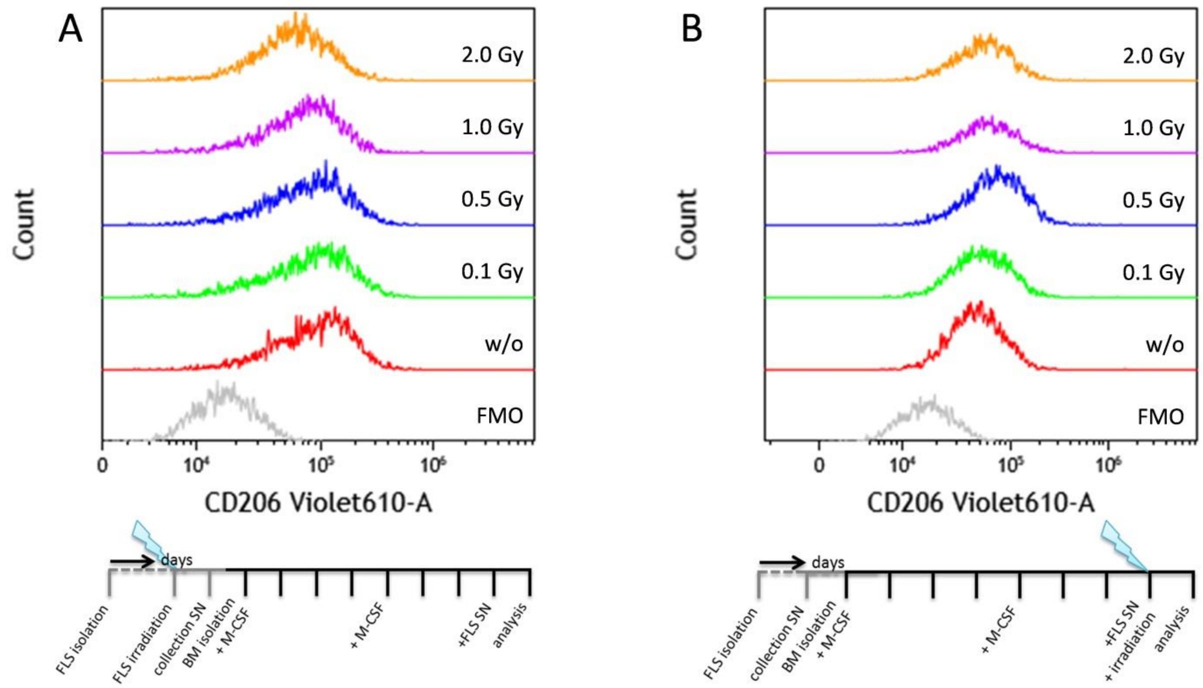

Supplementary Figure 4: Exemplary histograms of the flow cytometry-based analyses of expression of CD206 macrophage surface marker

Bone marrow cells from the long bones of h*TNFα* tg mice were isolated and differentiated into M0 macrophages. Prior to macrophage generation, fibroblast-like synoviocyte cultures (FLS) were generated, phenotyped and partly irradiated. Conditioned SN of irradiated and non-irradiated FLS cultures were collected and stored at -80°C until they were added to macrophage cultures 24hrs prior to characterisation of the macrophage phenotype. Shown is an exemplary experiment that visualizes the decreased surface expression of CD206 starting from 0.5Gy when macrophages are in contact with SN of irradiated FLS (A) and the slightly increased surface expression of CD206 when macrophages that had been in contact with SN of FLS are irradiated with 0.5Gy (B).
